# Tetrathiafulvalene-2,3,6,7-tetrathiolate linker redox-state elucidation *via* S K-edge X-ray absorption spectroscopy†

**DOI:** 10.1039/d3cc02325g

**Published:** 2023-06-29

**Authors:** Ningxin Jiang, Jan-Niklas Boyn, Arun Ramanathan, Henry S. La Pierre, John S. Anderson

**Affiliations:** a Department of Chemistry and The James Franck Institute, University of Chicago Chicago Illinois 60637 USA jsanderson@uchicago.edu; b Department of Mechanical and Aerospace Engineering, Princeton University Princeton New Jersey 08544-5263 USA; c School of Chemistry and Biochemistry, Georgia Institute of Technology Atlanta Georgia 30332-0400 USA hsl@gatech.edu

## Abstract

Sulfur K-edge XAS data provide a unique tool to examine oxidation states and covalency in electronically complex S-based ligands. We present sulfur K-edge X-ray absorption spectroscopy on a discrete redox-series of Ni-based tetrathiafulvalene tetrathiolate (TTFtt) complexes as well as on a 1D coordination polymer (CP), NiTTFtt. Experiment and theory suggest that Ni–S covalency decreases with oxidation which has implications for charge transport pathways.

## Abstract 2

S K-edge XAS data reveal decreasing covalency upon oxidation and a distinct signature for doubly oxidized TTF cores in NiTTFtt based complexes and materials.

## Graphical abstract

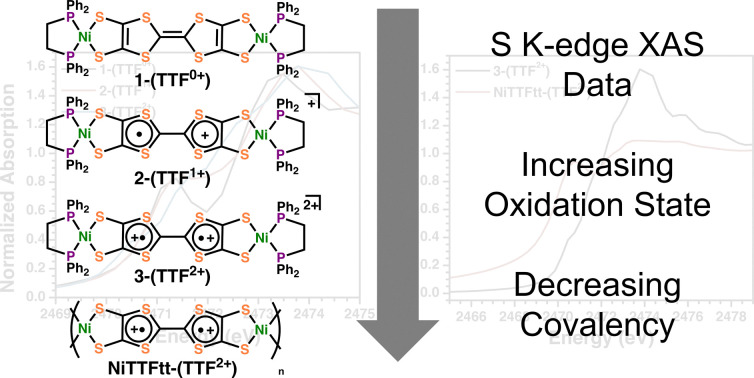

Dithiolene and tetrathiafulvalene (TTF)-based compounds have generated significant interest due to their remarkable properties.^1^ For instance, high electric conductivity has been observed in dithiolene-based CPs, such as Ni_3_(BHT)_2_ (BHT = benzenehexathiolate),^2^ Cu_3_(BHT),^3^ and cobalt dithiolenes,^4^ as well as in TTF-based metal organic frameworks.^5^ Some sulfur-based materials, such as (TTF)[Ni(dmit)_2_]_2_^6^ and (TTF)[Pd(dmit)_2_]_2_^7^ (dmit = 1,3-dithiole-2-thione-4,5-dithiolate), which possess both dithiolene motifs and TTF, are also superconductors.^8^

Tetrathiafulvalene-2,3,6,7-tetrathiolate (TTFtt) is a particularly attractive sulfur-based ligand for developing novel materials since it has features of both dithiolene and TTF cores.^9^ Exciting physical properties have been observed in TTFtt-based molecules and materials. For example, [(FeTPA)_2_TTFtt][BAr^F^_4_]_2_ exhibits reversible switching of diradical character *via* metal-centered spin crossover,^10^ Pt-based TTFtt molecules show bright, modular, and switchable near-infrared II emission,^11^ and NiTTFtt CPs display glassy-metallic conductivity.^12^

TTFtt can be oxidized twice leading to three distinct redox states which dramatically influence properties. The CP NiTTFtt has formally doubly oxidized TTFtt linkers with high conductivity and n-type carriers,^12^ but NiTTFtt^2−^, with neutral TTF cores, is a p-type semiconductor.^13^ While the formal redox state and charge for these materials is unambiguous, determining the localization of oxidation, namely between the linker and the metal center, is more challenging. Direct correlation between spectroscopic evidence and TTFtt oxidation state is lacking despite observed differences in vibrational, UV-visible, and near-infrared data between TTFtt redox isomers.^13^

X-ray absorption spectroscopy (XAS) enables experimental examination of oxidation states and covalency. In particular, S K-edge XAS enables analysis of the redox states of non-innocent S-based ligands in dithiolene compounds.^14,15^ For instance, in [Ni(S_2_C_2_Me_2_)_2_]^n−^, S K-edge XAS suggests that oxidation primarily occurs on the ligand as opposed to Ni.^16^ We therefore collected sulfur K-edge XAS data on a series of {[(dppe)Ni]_2_TTFtt}^n−^ molecules as well as a related CP. We also performed time-dependent density functional theory (TD-DFT) calculations to interpret this data. We find that the covalency of the metal sulfur bond decreases with higher TTFtt redox states with a characteristic peak for the doubly oxidized TTFtt-based moieties. Calculations suggest that this characteristic peak can be assigned to excitation into a π-based lowest unoccupied molecular orbital (LUMO). These results elucidate the electronic structures of TTFtt systems and provide insights into how these electronic structures are linked to the observed bulk properties in TTFtt-based materials.

Three previously reported Ni-based TTFtt molecules with different TTFtt redox states were chosen for XAS characterization (Fig. 1a).^9^ The complex [(dppe)Ni]_2_TTFtt (1-(TTF^0+^)) contains a neutral TTF, while {[(dppe)Ni]_2_TTFtt}{BAr^F^_4_} (2-(TTF^1+^)) has a singly-oxidized TTF and the TTF in {[(dppe)Ni]_2_TTFtt}{BAr^F^_4_}_2_ (3-(TTF^2+^)) is doubly oxidized (BAr^F^_4_ = tetrakis[3,5-bis(trifluoromethyl)phenyl]borate). Experimental S K-edge XAS data of these complexes are shown in Fig. 1b. Both 1-(TTF^0+^) and 2-(TTF^1+^) clearly display one pre-edge feature at 2471.5 eV. In contrast, 3-(TTF^2+^) displays three distinct pre-edge shoulders at ∼2470.5, 2471.5 and 2472.5 eV. Curve-fitting analyses were performed using pseudo-Voigt line shapes and a step function (Fig. 2, further details in ESI†).^17^ Fitting provides pre-edge peak positions of 2471.2 (1-(TTF^0+^)) and 2471.4 (2-(TTF^1+^)) eV. For 3-(TTF^2+^), three pre-edge peaks are fit at 2470.6, 2471.5, and 2472.5 eV. We note that fits with an additional pre-edge feature for 2-(TTF^1+^) were unsuccessful (see ESI†).

**Fig. 1:**
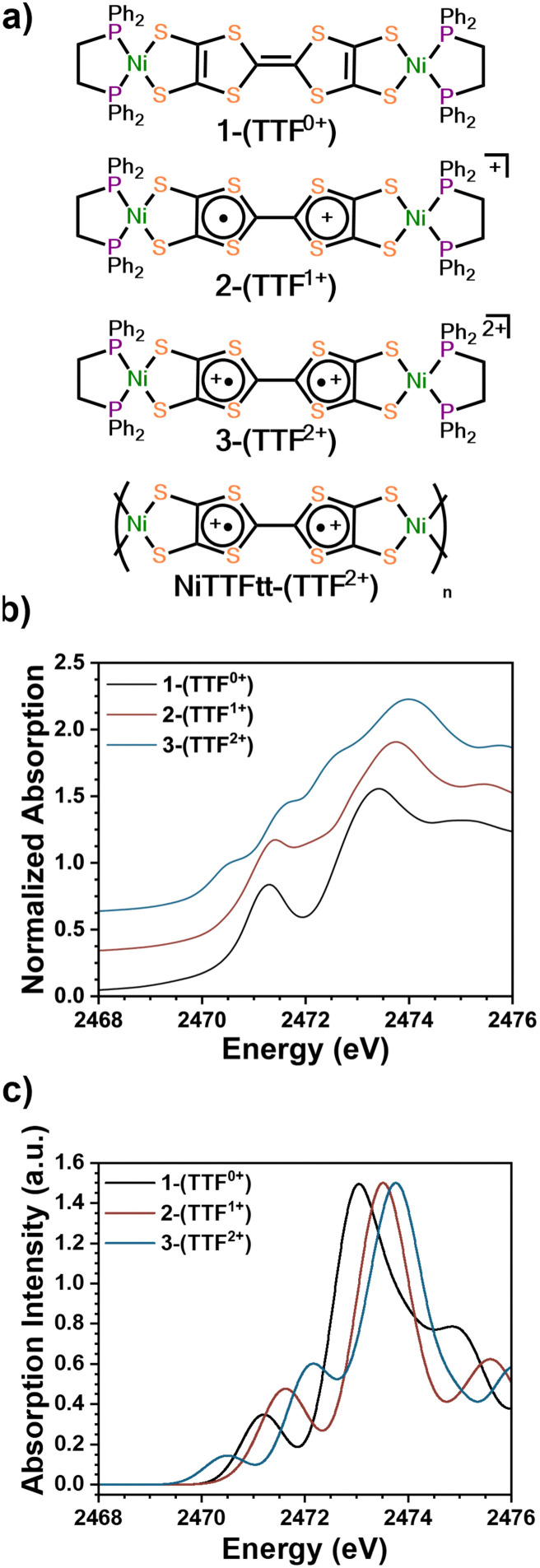
(a) The structure of 1-(TTF^0+^), 2-(TTF^1+^), and 3-(TTF^2+^). (b) Normalized experimental sulfur K-edge XAS spectra of 1-(TTF^0+^), 2-(TTF^1+^), and 3-(TTF^2+^). An offset of 0.3 was applied to each curve. (c) TD-DFT calculated sulfur K-edge XAS spectra of 1-(TTF^0+^), 2-(TTF^1+^), and 3-(TTF^2+^).

**Fig. 2:**
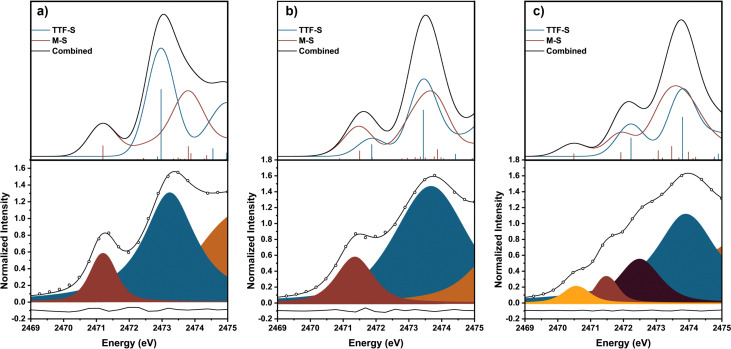
Experimental and TD-DFT calculated XAS sulfur K-edge spectra of (a) 1-(TTF^0+^), (b) 2-(TTF^1+^), and (c) 3-(TTF^2+^). The top spectra are the theory results from TD-DFT, and the bottom spectra are the experimental results. The sticks in the top figures represent the relative intensity of each transition based on calculated oscillator strengths. The blue sticks represent transitions from the core TTF S's and the red sticks represent contributions from the dithiolene S's bound to Ni. In the bottom spectra, the fitted peaks are shown with the residual shown in gray.

We used TD-DFT calculations to understand the pre-edge features in the XAS spectra (Fig. 1c and 2). Computations used the PBE0 functional^18^ with the zero-order regular approximation (ZORA) and the ZORA-def2-TZVP and SARC/J basis sets^19–21^ utilizing the Tamm–Dancoff approximation^22^ as implemented in Orca 5.0.3.^23^ We note that there are two distinct sets of S atoms in the TTFtt ligand: the four S's bound to Ni (M-S's) and the four S's located in the TTF core (TTF-S's). The TD-DFT calculations enable differentiation between these different S types.

Each calculated spectrum in Fig. 2 (black curve) is composed of M–S contributions (red curve) and the TTF–S contributions (blue curve). For 1-(TTF^0+^), the pre-edge feature at 2471.2 eV primarily arises from M–S based transitions. For 2-(TTF^1+^), the calculations suggest that the pre-edge feature at 2471.4 eV arises from both M–S and TTF–S based transitions. For 3-(TTF^2+^), the first feature at 2470.6 eV can be assigned to a M–S based transition, but assignment of the features at 2471.5 eV and 2472.5 eV is more complicated. One broader feature is found in the calculated spectrum (2472.2 eV), but examination of the constitutive transitions of this feature suggests that it is a convolution of two distinct features, similar to the pre-edge feature in 2-(TTF^1+^) (see below). The experimental spectrum evinces greater resolution of these features which may be due to multiplet effects not captured in the calculation. Based on the calculated and experimental peak intensities, the peak at 2471.5 eV is assigned to a M–S transition and the peak at 2472.5 eV primarily arises from TTF–S transitions (see ESI†).

The intensities of pre-edge peaks in ligand K-edge XAS data are proportional to the bond covalency.^16,17,24,25^ Since TD-DFT calculations allow for deconvolution of the M–S contributions, the Ni–S bond covalency can be determined. Calculations indicate that the pre-edge feature in 2-(TTF^1+^) at 2471.4 eV arises from both M–S and TTF–S based transitions which precludes assigning the fitted intensity to a single transition. For 1-(TTF^0+^), the Ni–S relative covalency of the peak at 2471.2 eV can be determined from the intensity of the peak which is 0.56(3). For 3-(TTF^2+^), the Ni–S covalency is from both peaks at 2470.6 and 2471.5 eV with a combined intensity of 0.47(5); smaller than in 1-(TTF^0+^). The lower value in 3-(TTF^2+^) suggests a lower bond covalency in 3-(TTF^2+^) and, therefore, a higher ionic character for the Ni–S bonds. DFT calculations also show a decrease of the Ni–S Mayer bond order from 0.888 in 1-(TTF^0+^) to 0.802 in 3-(TTF^2+^), and natural bond order (NBO) analysis^26^ reveals a more ionic S-localized bond with the S-based NBO percentage increasing from 69.9% in 1-(TTF^0+^) to 73.7% in 3-(TTF^2+^). All these data are consistent with decreasing covalency upon oxidation in these TTFtt complexes.

In previous Ni dithiolene S K-edge XAS data the hole-weighted covalencies were also found to decrease with higher ligand oxidation states which was rationalized with an inverted bonding description.^16^ In the present example, oxidation of the TTFtt increases S-bonding in the organic core of the linker, thereby decreasing covalency with Ni. An increase in C–S bonding covalency is supported by a similar increase in the computed C–S bond order (Table S2, ESI†). Thus, while similar trends with oxidation are observed between these TTFtt-based compounds and previous dithiolene examples, we propose that the origins of these effects are subtly different.

We note a distinctive peak at 2470.6 eV in 3-(TTF^2+^) which is not observed in 1-(TTF^0+^) and 2-(TTF^1+^). The natural transition orbitals (NTOs) of the XAS transitions are shown in Fig. 3 and Fig. S6 (ESI†) and provide insight into this feature. All three compounds have a feature between 2471–2472 eV which stems from a transition from the M–S S-based s-orbitals into an orbital localized on the respective half of the molecule. For 3-(TTF^2+^), the low energy peak at 2470.6 eV can be assigned to a transition from the M–S s-orbitals into the TTFtt π LUMO. This DFT-predicted peak is also present in 2-(TTF^1+^) but displays significantly lower intensity because this orbital is singly occupied in the monocationic complex. The increased intensity in 3-(TTF^2+^) provides a diagnostic signal for this formally doubly oxidized TTFtt oxidation state. A similar 2470.5 eV peak has also been observed in a TTF-BA (BA = bromanil, C_6_Br_4_O_2_) compound.^27^ As a final note, calculations predict that the TTF-S pre-edge transitions at ∼2472 eV also correspond to excitations into TTFtt-based π LUMO orbitals which are similarly only accessible in the oxidized compounds (Fig. S6, ESI†).

**Fig. 3:**
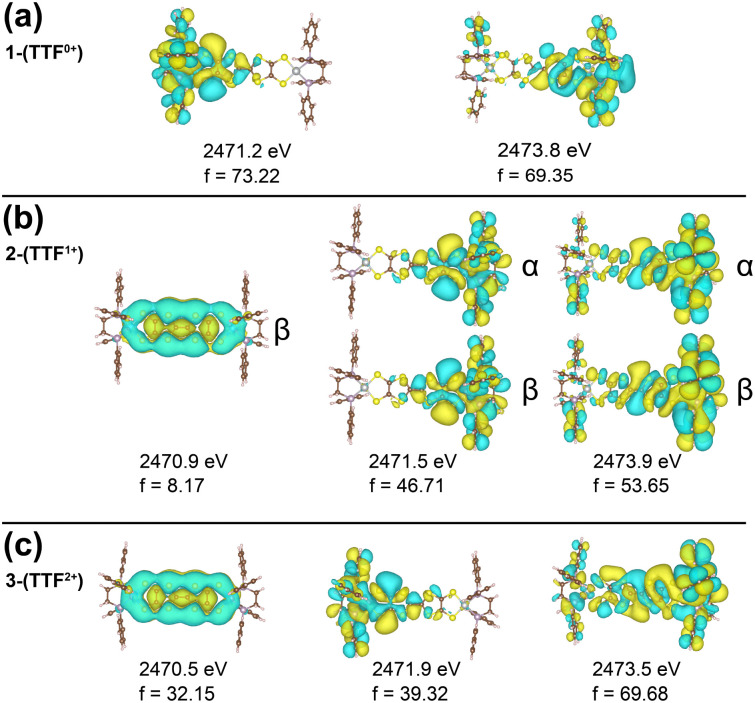
TD-DFT NTOs, energies, and intensities (f) for the dominant transitions from the M-S's contributing to (a) 1-(TTF^0+^), (b) 2-(TTF^1+^), and (c) 3-(TTF^2+^) sulfur K-edge XAS.

The S K-edge XAS data on the NiTTFtt-(TTF^2+^) CP which has formally doubly oxidized TTFtt linkers (Fig. 4) were also examined. This material exhibits glassy metallic conductivity, and more detailed understanding of its electronic structure and how it relates to its bulk properties is valuable.^12^ Unfortunately, the corresponding reduced CP with neutral TTFtt linkers, Li-NiTTFtt-(TTF^0+^), is too air-sensitive for reliable acquisition of XAS data.^13^NiTTFtt-(TTF^2+^) shows only one prominent pre-edge feature at ∼2470.7 eV which is very close to the position of the lowest energy pre-edge peak observed in 3-(TTF^2+^) suggesting similar formal TTFtt oxidation states in NiTTFtt-(TTF^2+^) and 3-(TTF^2+^). The peak position was determined from 2nd derivative analysis due to the trailing absorption in NiTTFtt-(TTF^2+^) which may be due to disordered sites and a band-like electronic structure as observed in MoS_2_ S K-edge data.^28^

**Fig. 4:**
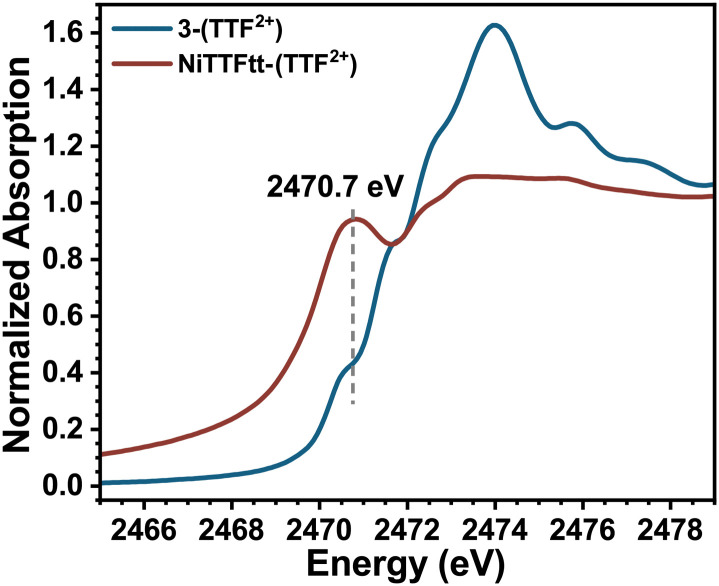
Experimental sulfur K-edge XAS results of 3-(TTF^2+^) (black curve) and NiTTFtt-(TTF^2+^) (red curve).

The structures of 1-(TTF^0+^) and 3-(TTF^2+^) are similar to the smallest repeat units of Li-NiTTFtt-(TTF^0+^) and NiTTFtt-(TTF^2+^) and thus the XAS data on these molecular compounds can provide insights on the properties of TTFtt-based CPs. Indeed, such dimensional reduction studies of molecular compounds have been performed on several extended solids.^29,30^ The electrical conductivity of NiTTFtt-(TTF^2+^) (470 S cm^−1^) is significantly higher than that of Li-NiTTFtt-(TTF^0+^) (10 S cm^−1^). Limiting charge transport pathways in CPs include hopping and band-like transport.^31^ For band-like transport, increasing the metal-ligand covalency should, in principle, increase conductivity.^32^ A trend of decreasing covalency upon oxidation from 1-(TTF^0+^) to 3-(TTF^2+^) is observed along with similar overall electronic structures for 3-(TTF^2+^) and NiTTFtt-(TTF^2+^). These observations suggest an apparent dichotomy, namely decreasing Ni–S covalency but increasing conductivity upon oxidation. Previous calculations on NiTTFTT-(TTF^2+^) suggest that the metallic character arises primarily from linker-linker interactions.^12^ Therefore the decreased Ni–S covalency may not be as important for bulk conductivity. We note that TD-DFT calculations predict that the 2470.5 eV pre-edge feature observed in 3-(TTF^2+^) originates from a transition into a TTFtt π-based LUMO. A similar feature in NiTTFTT-(TTF^2+^) suggests that similar π-based transitions are present, and may indicate better π-stacking interactions between TTFtt motifs in NiTTFtt-(TTF^2+^) as the source of enhanced conductivity.^31^

In conclusion, S K-edge XAS and TD-DFT studies were conducted on Ni-based TTFtt systems with different TTFtt redox states. The Ni–S bonding covalency decreases with higher TTFtt redox states. A unique feature is observed at ∼2470.5 eV for the doubly oxidized TTFtt linkers and can be assigned to a transition into a π-type orbital. Importantly, while Ni–S covalency decreases upon oxidation, conductivity increases, supporting that the major conductivity pathways in TTFtt based materials are through linker-linker interactions. Thus, the results presented here anchor formal TTFtt redox states with spectroscopic signatures and provide insights into the unusual charge transport behavior observed in TTFtt CPs.

This work was supported by the ARO (W911NF-20-1-0091). J. S. A. acknowledges support from the DOE (DE-SC0019215), and from the NSF (DMR-2002367). A. R. and H. S. L. acknowledge support for spectroscopic studies through NSF grant CHE-1943452. Use of the Stanford Synchrotron Radiation Lightsource, SLAC National Accelerator Laboratory, is supported by the U.S. DOE, BES (DE-AC02-76SF00515). The SSRL Structural Molecular Biology Program is supported by the DOE BER, and by the NIGMS (P41GM103393).

## Conflicts of interest

There are no conflicts to declare.

## Supplementary Material

CC-059-D3CC02325G-s001

## References

[cit1] Xie J., Wang L., Anderson J. S. (2020). Chem. Sci..

[cit2] Kambe T., Sakamoto R., Kusamoto T., Pal T., Fukui N., Hoshiko K., Shimojima T., Wang Z., Hirahara T., Ishizaka K. (2014). J. Am. Chem. Soc..

[cit3] Huang X., Sheng P., Tu Z., Zhang F., Wang J., Geng H., Zou Y., Di C.-A., Yi Y., Sun Y. (2015). Nat. Commun..

[cit4] Clough A. J., Skelton J. M., Downes C. A., De La Rosa A. A., Yoo J. W., Walsh A., Melot B. C., Marinescu S. C. (2017). J. Am. Chem. Soc..

[cit5] Park S. S. , Hontz E. R., Sun L., Hendon C. H., Walsh A., Van Voorhis T. and Dincă M., J. Am. Chem. Soc., 2015, **137**, 1774–1777 10.1021/ja512437u 25597934

[cit6] Brossard L., Ribault M., Bousseau M., Valade L., Cassoux P. (1986). C. R. Acad. Sci., Ser. II: Mec., Phys., Chim., Sci. Terre Univers.

[cit7] Brossard L., Hurdequint H., Ribault M., Valade L., Legros J., Cassoux P. (1988). Synth. Met..

[cit8] Kato R. (2004). Chem. Rev..

[cit9] Xie J., Boyn J.-N., Filatov A. S., McNeece A. J., Mazziotti D. A., Anderson J. S. (2020). Chem. Sci..

[cit10] Kawamura A., Xie J., Boyn J.-N., Jesse K. A., McNeece A. J., Hill E. A., Collins K. A., Valdez-Moreira J. A., Filatov A. S., Kurutz J. W. (2020). J. Am. Chem. Soc..

[cit11] McNamara L. E., Boyn J.-N., Melnychuk C., Anferov S. W., Mazziotti D. A., Schaller R. D., Anderson J. S. (2022). J. Am. Chem. Soc..

[cit12] Xie J., Ewing S., Boyn J.-N., Filatov A. S., Cheng B., Ma T., Grocke G. L., Zhao N., Itani R., Sun X. (2022). Nature.

[cit13] Xie J., Pan J.-A., Cheng B., Ma T., Filatov A. S., Patel S. N., Park J., Talapin D. V., Anderson J. S. (2022). J. Am. Chem. Soc..

[cit14] Eisenberg R., Gray H. B. (2011). Inorg. Chem..

[cit15] Sproules S., Wieghardt K. (2011). Coord. Chem. Rev..

[cit16] Szilagyi R. K., Lim B. S., Glaser T., Holm R. H., Hedman B., Hodgson K. O., Solomon E. I. (2003). J. Am. Chem. Soc..

[cit17] Solomon E. I., Hedman B., Hodgson K. O., Dey A., Szilagyi R. K. (2005). Coord. Chem. Rev..

[cit18] Perdew J. P., Ernzerhof M., Burke K. (1996). Chem. Phys..

[cit19] Van Lenthe E. v, Snijders J., Baerends E. (1996). Chem. Phys..

[cit20] Pantazis D. A., Chen X.-Y., Landis C. R., Neese F. (2008). J. Chem. Theory Comput..

[cit21] Neese F., Wennmohs F., Hansen A., Becker U. (2009). Chem. Phys..

[cit22] Hirata S., Head-Gordon M. (1999). Chem. Phys. Lett..

[cit23] Neese F., Wennmohs F., Becker U., Riplinger C. (2020). Chem. Phys..

[cit24] Minasian S. G., Keith J. M., Batista E. R., Boland K. S., Clark D. L., Conradson S. D., Kozimor S. A., Martin R. L., Schwarz D. E., Shuh D. K. (2012). J. Am. Chem. Soc..

[cit25] Lee K., Blake A. V., Donahue C. M., Spielvogel K. D., Bellott B. J., Daly S. R. (2019). Angew. Chem., Int. Ed..

[cit26] Foster J. P., Weinhold F. (1980). J. Am. Chem. Soc..

[cit27] Takahashi Y., Nakao H., Kumai R., Ishibashi S., Horiuchi S., Kohyama M., Kobayashi K., Yamasaki Y., Okamoto J., Sudayama T. (2014). J. Phys.: Conf. Ser..

[cit28] Guay D., Divigalpitiya W., Belanger D., Feng X. (1994). Chem. Mater..

[cit29] Yang L., Dincă M. (2021). Angew. Chem..

[cit30] Heinrich J. L., Berseth P. A., Long J. R. (1998). Chem. Commun..

[cit31] Xie L. S., Skorupskii G., Dincă M. (2020). Chem. Rev..

[cit32] Sun L., Hendon C. H., Minier M. A., Walsh A., Dincă M. (2015). J. Am. Chem. Soc..

