# Supplementary material for: Tetrathiafulvalene-2,3,6,7-tetrathiolate linker redox-state elucidation via S K-edge X-ray absorption spectroscopy
Source: Chem Commun (Camb). 2023 Jun 29;59(62):9537–40. doi: 10.1039/d3cc02325g (PMC10392963; doi:10.1039/d3cc02325g)
Supplement: CC-059-D3CC02325G-s001 [file CC-059-D3CC02325G-s001.pdf]

Electronic Supplementary Material (ESI) for ChemComm.
This journal is © The Royal Society of Chemistry 2023

Tetrathiafulvalene-2,3,6,7-tetrathiolate Linker Redox-State Elucidation via S K-edge X-ray Absorption Spectroscopy

Ningxin Jiang[a], Jan-Niklas Boyn[b], Arun Ramanathan[c], Henry S. La Pierre*[c,d,e], John S. Anderson*[a]

[a] Department of Chemistry and The James Franck Institute, University of Chicago, Chicago, Illinois, 60637, United States.

[b] Department of Mechanical and Aerospace Engineering and Andlinger Center for Energy and the Environment, Princeton University, Princeton, New Jersey, 08544-5263, United States.

[c] School of Chemistry and Biochemistry, Georgia Institute of Technology, Atlanta, Georgia, 30332-0400, United States.

[d] Nuclear and Radiological Engineering Program, Georgia Institute of Technology, Atlanta, Georgia, 30332-0400, United States.

[e] Physical Sciences Division, Pacific Northwest National Laboratory, Richland, Washington 99352, United States

Contents

1. Experimental section 3
2. XAS data analysis 4
3. Computational methodology 7
4. References 19

1. Experimental section

General considerations All manipulations were performed under an inert atmosphere of dry N_2 using a Schlenk line or MBraun UNIlab glovebox unless otherwise noted. Tetrahydrofuran (THF), diethyl ether (Et_2O) and acetonitrile (MeCN) used in preparing the materials were initially dried and purged with N_2 on a solvent purification system from Pure Process Technology. MeCN was then passed through activated alumina and stored over 4 Å molecular sieves. THF and Et_2O were stirred with liquid NaK alloy, filtered through activated alumina, and stored over 4 Å molecular sieves. All other chemicals were purchased from commercial sources and used as received unless noted. Elemental analyses (C, H, N) were performed by Midwest Microlabs.

Sample preparation The $(dppeNi)_2TTFtt$ (**1-(TTF^{0+})**), $[(dppeNi)_2TTFtt][BAr^F_4]$ (**2-(TTF^{1+})**), $[(dppeNi)_2TTFtt][BAr^F_4]_2$(**3-(TTF^{2+})**) (TTFtt = trathiafulvalene-2,3,6,7-tetrathiolate, BAr^F_4 = tetrakis[3,5-bis(trifluoromethyl)phenyl]borate) and NiTTFtt coordination polymer (CP) were synthesized as previously described.[1, 2]

X-ray absorption spectroscopy (XAS) sample preparation All four analytes, after drying under vacuum overnight, were ground with a mortar and pestle to yield a fine powder. An aluminum bracket with voids was used as sample holder with one side of the bracket covered with double-sided tape and the openings cut out and the other side covered with one sided sulfur-free tape. Inside a glovebox, the powder samples were painted as thinly as possible on the tape with a single-use paint brush. The backing of the double-sided tape was then removed and the sample was covered with a single layer of 3525 Ultralene® (0.16 mil). The samples were shipped to the line in sealed Mylar bags under nitrogen.

Sulfur K-edge XAS measurements Sulfur K-edge XAS data were measured at the Stanford Synchrotron Radiation Lightsource on the unfocused 20 pole, 2.0 T wiggler Beamline 4-3, under SPEAR3 storage ring parameters of 3 GeV and 500 mA. A Ni-coated, flat, bent premonochromator mirror was used for harmonic rejection and vertical collimation. A Si(111) double crystal monochromator was used for energy selection. A shutter was inserted automatically during each monochromator move to minimize photoreduction. The photon energy was calibrated to the maximum of the first pre-edge feature of $Na_2S_2O_3 \cdot 5H_2O$ at 2472.02 eV. At least three scans were measured for each sample to ensure reproducibility (and lack of beam degradation of the sample). Each sample was measured in duplicate.

2. XAS data analysis

All calibrations, background subtractions, normalizations, and averaging were performed using the Athena program in the IFFEFIT XAS software package as previously reported.[3] The background was removed from each spectrum by fitting a first order polynomial to the pre-edge region (2410 – 2450 eV). The data were normalized by fitting the post-edge region (2510 – 2690 eV) with a third order polynomial and setting the step function to an intensity of 1.0 at a set point of 2490 eV.

Fits to the spectra were performed with the IGOR Pro 9.0 software program using a modified version of EDGEFIT.[4] The data were modeled using pseudo-Voigt line shapes with a 1:1 Lorentzian/Gaussian mixture and a step function with a 1:1 arc-tangent and error function ratio. The number of curves and their positions were initially determined using the minima of the second derivative traces of the spectra. For both **1-(TTF^{0+})** and **2-(TTF^{1+})**, the second derivative clearly shows the number and position of the peaks. For **3-(TTF^{2+})**, two additional transitions, beyond those clearly observed in the second derivative analysis, are necessary to successfully model the pre-edge region. Curves were fit over a range 2465 to 2476 eV. The intensity of each pre-edge feature was determined by taking the product of the modeled amplitude and peak width at half-maximum.

Attempts to include an additional peak in the fit of **2-(TTF^{1+})** as hinted by visual inspection of the region between 2471.7 and 2472.5 eV, were unsuccessful. The additional peak position could not be constrained by analysis of the second derivative and further fitting attempts to include an additional peak were variable and unstable indicating insufficient experimental resolution to identify a second peak.

*Fitting attempts on **2-(TTF^{1+})***

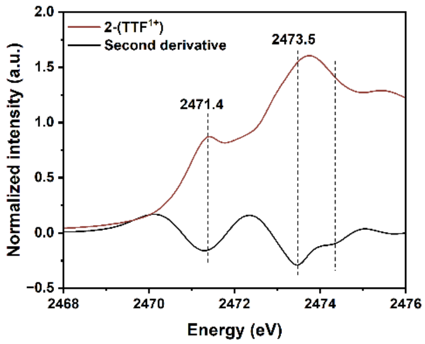

Figure S1. XAS data (red curve) and second derivative of **2-(TTF^{1+})** sulfur K-edge XAS (black curve).

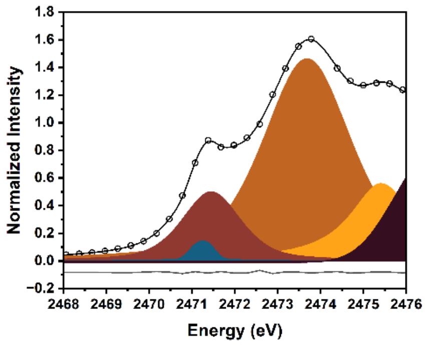

Figure S2. Fitting the **2-(TTF^{1+})** XAS spectrum to two-peak models in the pre-edge region with no restriction on the 2471.4 eV peak position.

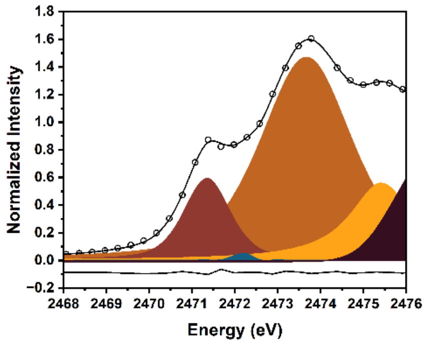

Figure S3. Fitting the **2-(TTF^{1+})** XAS spectrum to two-peak models in the pre-edge region with a restriction on the 2471.4 eV peak position.

Table S1. Summary of experimental peak positions, intensities of each peak and calculated Ni-S bond order of **1-(TTF^{0+})**, **2-(TTF^{1+})** and **3-(TTF^{2+})**

Compound	**1-(TTF^{0+})**	**2-(TTF^{1+})**	**3-(TTF^{2+})**
Experimental Ni-S bond length (Å)	N.A.	2.162	2.168
Calculated Ni-S bond length (Å)	2.182	2.194	2.213
Fitting parameters			
Step function position (eV)	2474.0(2)	2475.7(11)	2475.0(8)

Peak 1 position (eV)[a]	2471.2	2471.4	2470.6
Peak 1 amplitude	0.592(23)	0.579(17)	0.213(26)
Peak 1 FWHM	0.942(43)	1.31(4)	0.950(75)
Peak 1, intensity of peak	0.558(34)	0.759(31)	0.203(30)
Peak 2 position (eV)[a]	2473.2	2473.7	2471.5
Peak 2 amplitude	1.31(20)	1.47(23)	0.345(38)
Peak 2 FWHM	0.952(23)	2.59(15)	0.782(71)
Peak 2, intensity of peak	2.49(39)	3.80(63)	0.270(40)
Peak 3 position (eV)[a]		2475.4(4)	2472.5
Peak 3 amplitude		0.560(96)	0.553(72)
Peak 3 FWHM		1.75(31)	1.47(14)
Peak 4 position (eV)[a]			2473.9
Peak 4 amplitude			1.12(14)
Peak 4 FWHM			2.26(13)

[a.] The estimated standard deviations (esd) of most peak positions are not listed since these esd are smaller than 0.1 eV.

3. Computational methodology

Geometries were optimized at the PBE0[5]/def2-SVP[6] level of theory as implemented in Gaussian 16 Rev. A.03[7]. The sulfur K-edge absorption XAS data were then modelled using time-dependent density functional theory (TD-DFT) as implemented in ORCA version 5.0.3.[8] The PBE0 functional was used in combination with the zeroth-order regular approximation (ZORA)[9] to account for relativistic effects and the Tamm-Dancoff approximation (TDA).[10] The relativistically recontracted ZORA-Def2-TZVP[11] basis set was utilized in combination with the segmented all-electron relativistically contracted (SARC/J)[11] auxilliary basis set and the RIJCOSX[12] approximation. To facilitate comparison with experimental results, the effects of spectral broadening are illustrated in Figure S4, which shows the spectra using broadening of 1 eV, 1.5 eV and 2 eV. A broadening of 1 eV was selected for the discussion in the main text and the supporting information due to the good match between the results and the experimental spectra.

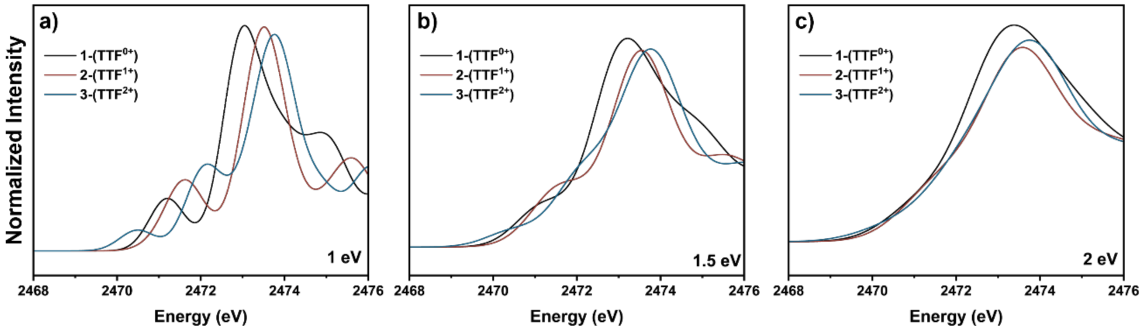

Figure S4. The TD-DFT calculated spectra of **1-(TTF^{0+})**, **2-(TTF^{1+})** and **3-(TTF^{2+})** with broadening of (a) 1eV, (b) 1.5 eV, and (c) 2 eV.

Peak assignment in 3-(TTF^{2+}). As mentioned in the main text, the assignment for the features in **3-(TTF^{2+})** at ~2471-2473 eV are convoluted since only one peak is apparent in the broadened calculated spectrum (2472.2 eV). The calculated TD-DFT spectrum is composed of individual M-S and TTF-S contributions, and each contribution is composed of multiple transitions. The sticks in the top of Figure 2 in the main text represent the relative intensities for each specific transition which compose the overall absorption features. The blue sticks represent transitions from the TTF-S contributions and the red sticks represent transitions from the M-S contributions. The calculation results suggest that the peak at 2472 eV arises from two different but closely spaced transitions. Calculations predict that the first transition at 2471.9 eV arises from M-S contributions and the second transition at 2472.2 eV can be assigned to a TTF-S contribution. These two calculated peak positions are very close to each other and are also more similar in energy to each other than the experimentally observed peaks (2471.5 eV and 2472.5 eV). While the overall predicted energies of these transitions are still in good agreement with experiment (the discrepancy in the individual features is < 0.5 eV), the convolution in the computed spectrum results in only one peak. We therefore used the relative intensities of these transitions to provide more evidence for peak assignments. In the experimental results, the intensity of peak 2471.5 eV is 0.27 while the intensity of the peak 2472.5 eV is 0.81 resulting in a rough ratio of 1:3. If the calculation results are instead used, the relative intensity of the dominant contribution to the transition at 2471.9 eV is 39.3 while the relative intensity of the dominant contribution the transition

at 2472.2 eV is 111, again for a ratio of ~1:3. Thus, the calculated intensities match well with the experimental intensities of the peaks if the peak at 2471.5 eV is assigned to the M-S contribution and the peak at 2472.5 eV is assigned to TTF-S contribution. We therefore propose that the experimentally observed peak at 2471.5 arises from M-S contributions and that the peak at 2472.5 arises from TTF-S contributions.

Table S2 TD-DFT NBO analysis and Mayer bond order of selected bond in **1-(TTF^{0+})**, **2-(TTF^{1+})** and **3-(TTF^{2+})**.

Compound	Ni-S MBO	Ni-S NBO %S	C-S$_a$ MBO	C-S$_a$ NBO %S	C-S$_b$ MBO	C-S$_b$ NBO %S	C-S$_c$ MBO	C-S$_c$ NBO %S
**1-(TTF^{0+})**	0.888	69.93	1.118	43.98	1.085	46.44	1.141	45.51
**2-(TTF^{1+})**	0.868	71.55	1.193	42.27	1.139	47.27	1.235	44.52
**3-(TTF^{2+})**	0.802	73.7	1.297	41.23	1.202	47.34	1.279	44.18

* MBO = Mayer bond order, NBO = natural bond orbitals, the %S stands for the contribution of sulfur to the NBO. The labels of C-S bonds are demonstrated in Figure S5.

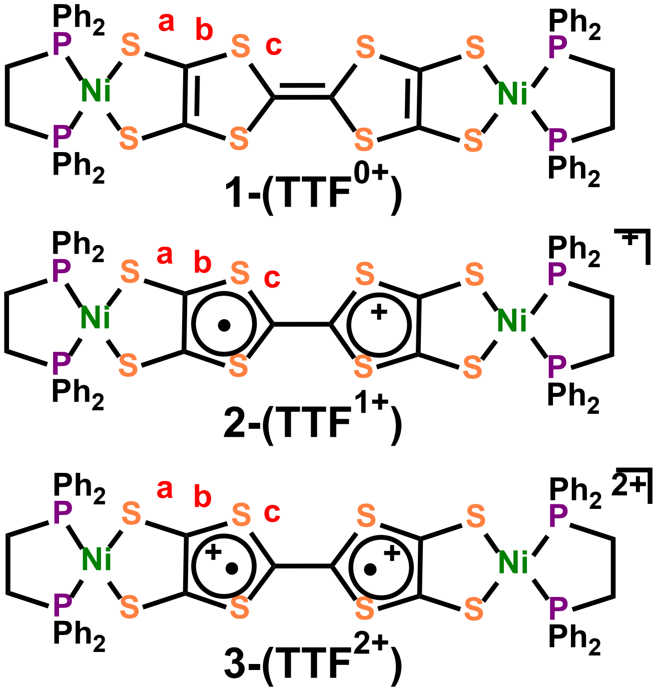

Figure S5. Demonstration of C-C bond labeling for compound **1-(TTF^{0+})**, **2-(TTF^{1+})** and **3-(TTF^{2+})**. Three different types of C-S bond was marked as C-S$_a$,C-S$_b$ and C-S$_c$.

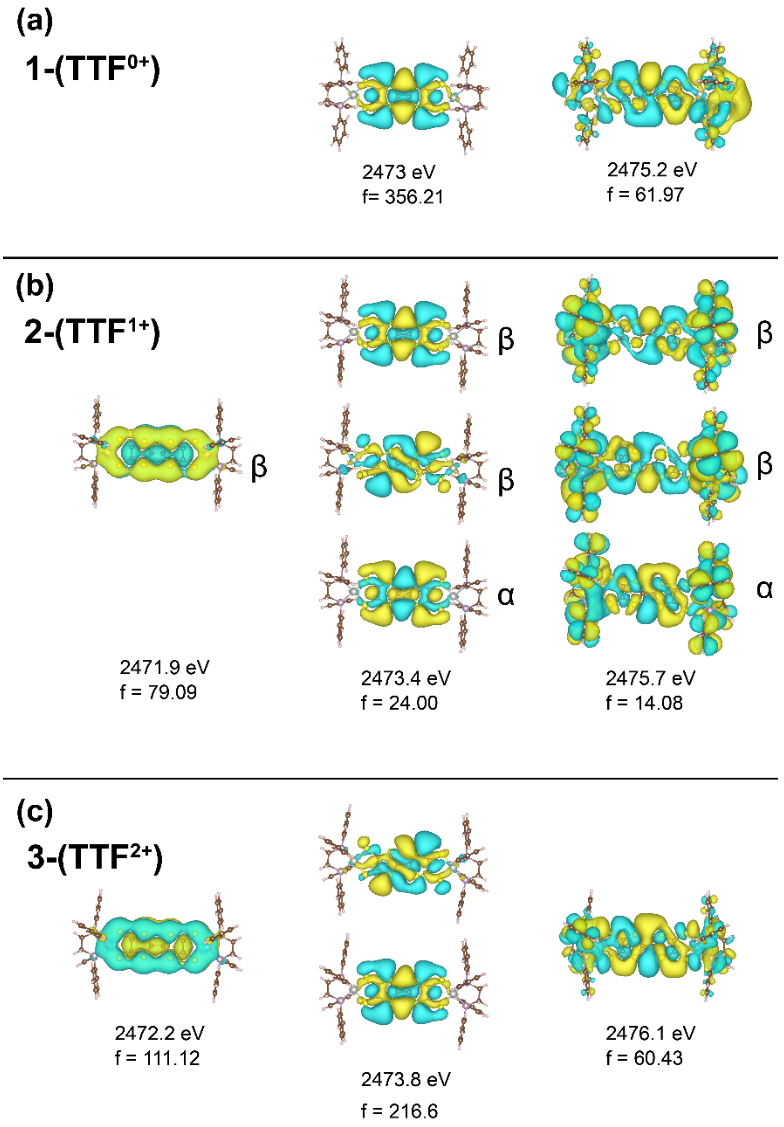

Figure S6. TD-DFT NTOs for dominant TTF-S transitions contributing to (a)**1-(TTF^{0+})**, (b)**2-(TTF^{1+})**, and (c)**3-(TTF^{2+})** sulfur K-edge XAS results.

Table S3. Atomic coordinates for the optimized structures of **1-(TTF^{0+})**, **2-(TTF^{1+})**, **3-(TTF^{2+})** from DFT calculations.

Compounds	Atom type	X (angstroms)	Y (angstroms)	Z (angstroms)
**1-(TTF^{0+})**	Ni	2.288062	3.23516	4.720287
	S	0.267894	2.608945	4.182879
	S	3.201933	2.295131	2.975744
	S	1.970335	0.757603	0.653811
	S	-0.75984	0.952348	1.843726

	P	1.622458	3.751116	6.720682
	P	4.108424	4.353739	5.10244
	C	0.251173	0.354811	0.51823
	C	1.804517	1.627907	2.177474
	C	0.566018	1.722101	2.713245
	C	-2.55249	5.573323	7.469602
	H	-3.53564	6.019932	7.63781
	C	-1.99853	4.72595	8.428758
	H	-2.54581	4.50618	9.348677
	C	-0.74866	4.148811	8.214031
	H	-0.32378	3.475842	8.963008
	C	-0.04392	4.421896	7.035749
	C	-0.61039	5.261917	6.06752
	H	-0.08312	5.437851	5.126211
	C	-1.85855	5.839068	6.289968
	H	-2.29759	6.487273	5.527959
	C	2.757228	5.056167	7.385256
	H	2.392003	6.003186	6.95446
	H	2.680234	5.154901	8.479111
	C	4.170598	4.750514	6.911133
	H	4.560439	3.838946	7.393657
	H	4.885335	5.557732	7.134142
	C	1.800111	2.319712	7.848393
	C	1.331801	1.079124	7.390452
	H	0.894462	1.00845	6.388677
	C	1.423677	-0.04738	8.204153
	H	1.051132	-1.007	7.837989
	C	1.998542	0.04551	9.471525
	H	2.079	-0.8422	10.10383
	C	2.475852	1.272735	9.92866
	H	2.929445	1.35133	10.91977
	C	2.374596	2.40674	9.12334
	H	2.747662	3.359987	9.504725
	C	4.067429	5.96966	4.242476
	C	3.66483	5.972368	2.89862
	H	3.383069	5.027153	2.422176
	C	3.626124	7.165917	2.182046
	H	3.315359	7.154414	1.134647
	C	3.968879	8.368529	2.799759
	H	3.927585	9.30506	2.238047
	C	4.359308	8.374697	4.137824
	H	4.6267	9.314102	4.627977
	C	4.412321	7.180865	4.856619

	H	4.728826	7.207637	5.901803
	C	5.756066	3.653183	4.754534
	C	5.940457	2.276804	4.94313
	H	5.090076	1.648141	5.21992
	C	7.195741	1.70775	4.740907
	H	7.3309	0.632953	4.882804
	C	8.268206	2.502692	4.338941
	H	9.249916	2.052432	4.172029
	C	8.084908	3.870169	4.135679
	H	8.920829	4.49331	3.808572
	C	6.832752	4.446079	4.340168
	H	6.690846	5.515924	4.167889
	S	-0.26789	-2.60895	-4.18288
	S	-3.20193	-2.29513	-2.97574
	S	-1.97034	-0.7576	-0.65381
	S	0.75984	-0.95235	-1.84373
	C	-0.25117	-0.35481	-0.51823
	C	-1.80452	-1.62791	-2.17747
	C	-0.56602	-1.7221	-2.71325
	Ni	-2.28806	-3.23516	-4.72029
	P	-1.62246	-3.75112	-6.72068
	P	-4.10842	-4.35374	-5.10244
	C	2.552487	-5.57332	-7.4696
	H	3.535641	-6.01993	-7.63781
	C	1.998525	-4.72595	-8.42876
	H	2.545806	-4.50618	-9.34868
	C	0.748658	-4.14881	-8.21403
	H	0.323779	-3.47584	-8.96301
	C	0.043921	-4.4219	-7.03575
	C	0.610392	-5.26192	-6.06752
	H	0.083122	-5.43785	-5.12621
	C	1.858546	-5.83907	-6.28997
	H	2.297592	-6.48727	-5.52796
	C	-2.75723	-5.05617	-7.38526
	H	-2.392	-6.00319	-6.95446
	H	-2.68023	-5.1549	-8.47911
	C	-4.1706	-4.75051	-6.91113
	H	-4.56044	-3.83895	-7.39366
	H	-4.88534	-5.55773	-7.13414
	C	-1.80011	-2.31971	-7.84839
	C	-1.3318	-1.07912	-7.39045
	H	-0.89446	-1.00845	-6.38868
	C	-1.42368	0.047376	-8.20415

	H	-1.05113	1.007004	-7.83799
	C	-1.99854	-0.04551	-9.47153
	H	-2.079	0.842197	-10.1038
	C	-2.47585	-1.27274	-9.92866
	H	-2.92945	-1.35133	-10.9198
	C	-2.3746	-2.40674	-9.12334
	H	-2.74766	-3.35999	-9.50473
	C	-4.06743	-5.96966	-4.24248
	C	-3.66483	-5.97237	-2.89862
	H	-3.38307	-5.02715	-2.42218
	C	-3.62612	-7.16592	-2.18205
	H	-3.31536	-7.15441	-1.13465
	C	-3.96888	-8.36853	-2.79976
	H	-3.92759	-9.30506	-2.23805
	C	-4.35931	-8.3747	-4.13782
	H	-4.6267	-9.3141	-4.62798
	C	-4.41232	-7.18087	-4.85662
	H	-4.72883	-7.20764	-5.9018
	C	-5.75607	-3.65318	-4.75453
	C	-5.94046	-2.2768	-4.94313
	H	-5.09008	-1.64814	-5.21992
	C	-7.19574	-1.70775	-4.74091
	H	-7.3309	-0.63295	-4.8828
	C	-8.26821	-2.50269	-4.33894
	H	-9.24992	-2.05243	-4.17203
	C	-8.08491	-3.87017	-4.13568
	H	-8.92083	-4.49331	-3.80857
	C	-6.83275	-4.44608	-4.34017
	H	-6.69085	-5.51592	-4.16789
**2-(TTF^{1+})**	Ni	2.280741	3.224468	4.704597
	S	0.250886	2.564872	4.196537
	S	3.20506	2.320094	2.932132
	S	1.936983	0.791949	0.645795
	S	-0.7268	0.918462	1.850487
	P	1.614699	3.76644	6.71045
	P	4.114707	4.334752	5.109284
	C	0.257093	0.363348	0.530313
	C	1.811289	1.639666	2.166592
	C	0.559679	1.711658	2.724037
	C	-2.62114	5.503072	7.267539
	H	-3.62102	5.927109	7.386465
	C	-2.22111	4.428703	8.059451

	H	-2.90639	4.006369	8.797987
	C	-0.94652	3.883644	7.911029
	H	-0.64265	3.036946	8.530384
	C	-0.06355	4.419618	6.967561
	C	-0.47858	5.485666	6.155772
	H	0.188379	5.883618	5.385245
	C	-1.74925	6.030127	6.314204
	H	-2.0654	6.86261	5.681512
	C	2.754106	5.070663	7.362372
	H	2.422669	6.024461	6.91969
	H	2.666748	5.17443	8.454355
	C	4.166483	4.721799	6.918153
	H	4.527658	3.810372	7.422534
	H	4.889932	5.520751	7.139697
	C	1.822892	2.347675	7.83877
	C	1.800734	1.054629	7.29846
	H	1.650834	0.923271	6.223218
	C	1.950509	-0.05399	8.130207
	H	1.928449	-1.05826	7.700827
	C	2.129454	0.118176	9.501422
	H	2.250314	-0.7517	10.1515
	C	2.15318	1.402745	10.04588
	H	2.28979	1.540752	11.121
	C	1.999969	2.514014	9.220394
	H	2.00977	3.512037	9.665745
	C	4.055839	5.958073	4.278011
	C	3.265655	6.092372	3.128215
	H	2.713226	5.22731	2.750884
	C	3.201042	7.315399	2.462408
	H	2.585329	7.409913	1.564983
	C	3.916188	8.411753	2.940362
	H	3.861554	9.370692	2.419444
	C	4.704323	8.285209	4.084885
	H	5.268951	9.142009	4.459976
	C	4.776121	7.064663	4.751912
	H	5.407537	6.981093	5.640043
	C	5.730996	3.591815	4.728548
	C	5.98464	2.300501	5.21423
	H	5.218191	1.764953	5.782274
	C	7.206956	1.686469	4.959252
	H	7.400049	0.682202	5.343235
	C	8.17562	2.346222	4.202111
	H	9.132481	1.860366	3.996963

	C	7.918281	3.619379	3.697854
	H	8.670376	4.132866	3.094451
	C	6.698566	4.243261	3.956273
	H	6.501084	5.238343	3.551719
	S	-0.25089	-2.56487	-4.19654
	S	-3.20506	-2.32009	-2.93213
	S	-1.93698	-0.79195	-0.6458
	S	0.726795	-0.91846	-1.85049
	C	-0.25709	-0.36335	-0.53031
	C	-1.81129	-1.63967	-2.16659
	C	-0.55968	-1.71166	-2.72404
	Ni	-2.28074	-3.22447	-4.7046
	P	-1.6147	-3.76644	-6.71045
	P	-4.11471	-4.33475	-5.10928
	C	2.621138	-5.50307	-7.26754
	H	3.621023	-5.92711	-7.38647
	C	2.221108	-4.4287	-8.05945
	H	2.906392	-4.00637	-8.79799
	C	0.946521	-3.88364	-7.91103
	H	0.642652	-3.03695	-8.53038
	C	0.063545	-4.41962	-6.96756
	C	0.478577	-5.48567	-6.15577
	H	-0.18838	-5.88362	-5.38525
	C	1.749245	-6.03013	-6.3142
	H	2.065395	-6.86261	-5.68151
	C	-2.75411	-5.07066	-7.36237
	H	-2.42267	-6.02446	-6.91969
	H	-2.66675	-5.17443	-8.45436
	C	-4.16648	-4.7218	-6.91815
	H	-4.52766	-3.81037	-7.42253
	H	-4.88993	-5.52075	-7.1397
	C	-1.82289	-2.34768	-7.83877
	C	-1.80073	-1.05463	-7.29846
	H	-1.65083	-0.92327	-6.22322
	C	-1.95051	0.053994	-8.13021
	H	-1.92845	1.058263	-7.70083
	C	-2.12945	-0.11818	-9.50142
	H	-2.25031	0.751703	-10.1515
	C	-2.15318	-1.40275	-10.0459
	H	-2.28979	-1.54075	-11.121
	C	-1.99997	-2.51401	-9.22039
	H	-2.00977	-3.51204	-9.66575
	C	-4.05584	-5.95807	-4.27801

	C	-3.26566	-6.09237	-3.12822
	H	-2.71323	-5.22731	-2.75088
	C	-3.20104	-7.3154	-2.46241
	H	-2.58533	-7.40991	-1.56498
	C	-3.91619	-8.41175	-2.94036
	H	-3.86155	-9.37069	-2.41944
	C	-4.70432	-8.28521	-4.08489
	H	-5.26895	-9.14201	-4.45998
	C	-4.77612	-7.06466	-4.75191
	H	-5.40754	-6.98109	-5.64004
	C	-5.731	-3.59182	-4.72855
	C	-5.98464	-2.3005	-5.21423
	H	-5.21819	-1.76495	-5.78227
	C	-7.20696	-1.68647	-4.95925
	H	-7.40005	-0.6822	-5.34324
	C	-8.17562	-2.34622	-4.20211
	H	-9.13248	-1.86037	-3.99696
	C	-7.91828	-3.61938	-3.69785
	H	-8.67038	-4.13287	-3.09445
	C	-6.69857	-4.24326	-3.95627
	H	-6.50108	-5.23834	-3.55172
**3-(TTF^{2+})**	Ni	2.28326	3.223362	4.710968
	S	0.259499	2.457961	4.246202
	S	3.186549	2.404631	2.863987
	S	1.90081	0.881301	0.612951
	S	-0.68423	0.835396	1.897228
	P	1.619054	3.801398	6.722313
	P	4.13181	4.325684	5.143323
	C	0.261188	0.368528	0.538918
	C	1.818592	1.679299	2.144287
	C	0.557804	1.674081	2.758937
	C	-2.65945	5.453849	7.152592
	H	-3.6716	5.854068	7.246505
	C	-2.3408	4.22366	7.72377
	H	-3.10177	3.655847	8.263781
	C	-1.04881	3.711921	7.613015
	H	-0.80696	2.747441	8.064867
	C	-0.06607	4.439884	6.931312
	C	-0.39707	5.667909	6.337487
	H	0.350183	6.235705	5.775157
	C	-1.68722	6.174336	6.457053
	H	-1.93689	7.135979	6.00321

	C	2.763481	5.100947	7.365061
	H	2.465317	6.061953	6.915699
	H	2.658676	5.202376	8.455823
	C	4.173643	4.71418	6.948672
	H	4.51109	3.809294	7.479342
	H	4.906997	5.508266	7.155219
	C	1.832753	2.39425	7.854448
	C	2.086225	1.120438	7.329266
	H	2.120852	0.976391	6.245879
	C	2.273623	0.036233	8.185747
	H	2.469162	-0.95532	7.771311
	C	2.209384	0.217511	9.565787
	H	2.356957	-0.63238	10.23626
	C	1.952857	1.483872	10.09475
	H	1.896901	1.625265	11.17647
	C	1.765116	2.570455	9.245309
	H	1.553433	3.553959	9.673839
	C	4.071786	5.947689	4.322767
	C	3.08935	6.194292	3.355035
	H	2.389571	5.401445	3.076976
	C	3.017683	7.440703	2.734101
	H	2.250985	7.627535	1.978826
	C	3.923058	8.443495	3.074938
	H	3.865912	9.420044	2.588412
	C	4.90707	8.201966	4.035508
	H	5.620537	8.986101	4.298825
	C	4.98386	6.960073	4.65938
	H	5.767144	6.781561	5.401231
	C	5.721033	3.550183	4.738249
	C	6.106203	2.399258	5.443403
	H	5.470017	1.989203	6.233393
	C	7.30613	1.76457	5.139022
	H	7.607103	0.876052	5.698498
	C	8.12075	2.260785	4.120083
	H	9.063301	1.761644	3.883689
	C	7.730962	3.389703	3.402612
	H	8.364624	3.776424	2.601352
	C	6.53263	4.034531	3.705158
	H	6.2353	4.919788	3.13874
	S	-0.2595	-2.45796	-4.2462
	S	-3.18655	-2.40463	-2.86399
	S	-1.90081	-0.8813	-0.61295
	S	0.684225	-0.8354	-1.89723

	C	-0.26119	-0.36853	-0.53892
	C	-1.81859	-1.6793	-2.14429
	C	-0.5578	-1.67408	-2.75894
	Ni	-2.28326	-3.22336	-4.71097
	P	-1.61905	-3.8014	-6.72231
	P	-4.13181	-4.32568	-5.14332
	C	2.659452	-5.45385	-7.15259
	H	3.671595	-5.85407	-7.24651
	C	2.340795	-4.22366	-7.72377
	H	3.101765	-3.65585	-8.26378
	C	1.048805	-3.71192	-7.61302
	H	0.806961	-2.74744	-8.06487
	C	0.066065	-4.43988	-6.93131
	C	0.397073	-5.66791	-6.33749
	H	-0.35018	-6.23571	-5.77516
	C	1.687224	-6.17434	-6.45705
	H	1.936888	-7.13598	-6.00321
	C	-2.76348	-5.10095	-7.36506
	H	-2.46532	-6.06195	-6.9157
	H	-2.65868	-5.20238	-8.45582
	C	-4.17364	-4.71418	-6.94867
	H	-4.51109	-3.80929	-7.47934
	H	-4.907	-5.50827	-7.15522
	C	-1.83275	-2.39425	-7.85445
	C	-2.08623	-1.12044	-7.32927
	H	-2.12085	-0.97639	-6.24588
	C	-2.27362	-0.03623	-8.18575
	H	-2.46916	0.95532	-7.77131
	C	-2.20938	-0.21751	-9.56579
	H	-2.35696	0.632375	-10.2363
	C	-1.95286	-1.48387	-10.0947
	H	-1.8969	-1.62527	-11.1765
	C	-1.76512	-2.57046	-9.24531
	H	-1.55343	-3.55396	-9.67384
	C	-4.07179	-5.94769	-4.32277
	C	-3.08935	-6.19429	-3.35504
	H	-2.38957	-5.40145	-3.07698
	C	-3.01768	-7.4407	-2.7341
	H	-2.25099	-7.62754	-1.97883
	C	-3.92306	-8.4435	-3.07494
	H	-3.86591	-9.42004	-2.58841
	C	-4.90707	-8.20197	-4.03551
	H	-5.62054	-8.9861	-4.29883

	C	-4.98386	-6.96007	-4.65938
	H	-5.76714	-6.78156	-5.40123
	C	-5.72103	-3.55018	-4.73825
	C	-6.1062	-2.39926	-5.4434
	H	-5.47002	-1.9892	-6.23339
	C	-7.30613	-1.76457	-5.13902
	H	-7.6071	-0.87605	-5.6985
	C	-8.12075	-2.26079	-4.12008
	H	-9.0633	-1.76164	-3.88369
	C	-7.73096	-3.3897	-3.40261
	H	-8.36462	-3.77642	-2.60135
	C	-6.53263	-4.03453	-3.70516
	H	-6.2353	-4.91979	-3.13874

4. References

1. J. Xie, J.-N. Boyn, A. S. Filatov, A. J. McNeece, D. A. Mazziotti and J. S. Anderson, *Chem. Sci.*, 2020, **11**, 1066-1078.
2. J. Xie, S. Ewing, J.-N. Boyn, A. S. Filatov, B. Cheng, T. Ma, G. L. Grocke, N. Zhao, R. Itani and X. Sun, *Nature*, 2022, 1-6.
3. B. Ravel and M. Newville, *J. Synchrotron Radiat.*, 2005, **12**, 537-541.
4. E. I. Solomon, B. Hedman, K. O. Hodgson, A. Dey and R. K. Szilagyi, *Coord. Chem. Rev.*, 2005, **249**, 97-129.
5. J. P. Perdew, M. Ernzerhof and K. Burke, *Chem. Phys.*, 1996, **105**, 9982-9985.
6. F. Weigend and R. Ahlrichs, *Phys. Chem. Chem. Phys.*, 2005, **7**, 3297-3305.
7. M. e. Frisch, G. Trucks, H. B. Schlegel, G. Scuseria, M. Robb, J. Cheeseman, G. Scalmani, V. Barone, G. Petersson and H. Nakatsuji, *Journal*, 2016.
8. F. Neese, F. Wennmohs, U. Becker and C. Riplinger, *Chem. Phys.*, 2020, **152**, 224108.
9. E. v. Van Lenthe, J. Snijders and E. Baerends, *Chem. Phys.*, 1996, **105**, 6505-6516.
10. S. Hirata and M. Head-Gordon, *Chem. Phys. Lett.*, 1999, **314**, 291-299.
11. D. A. Pantazis, X.-Y. Chen, C. R. Landis and F. Neese, *J. Chem. Theory Comput.*, 2008, **4**, 908-919.
12. F. Neese, F. Wennmohs, A. Hansen and U. Becker, *Chem. Phys.*, 2009, **356**, 98-109.
